# Asthma in the elderly patient

**DOI:** 10.1186/s40733-015-0015-1

**Published:** 2016-02-02

**Authors:** Louis-Philippe Boulet

**Affiliations:** Institut universitaire de cardiologie et de pneumologie de Québec, Université Laval, 2725, Chemin Sainte-Foy, Québec City, Québec G1V 4G5 Canada

**Keywords:** Asthma, Elderly, Asthma control, Asthma treatment, Aging

## Abstract

Asthma affects a significant proportion of elderly patients, but unfortunately, it is responsible for a high asthma-related morbidity and mortality in this population. This may be related not only to the development of a more severe asthma phenotype compared to younger patients, with more marked airway obstruction and a more neutrophilic type of airway inflammation, but also to the presence of many co-morbid conditions. Furthermore, in older patients, asthma is often under-diagnosed, undertreated and poorly managed. Unfortunately, elderly patients have usually been excluded of clinical trials on asthma and there is an urgent need to perform more research on the optimal management of asthma in this population.

## Introduction

### Case report

A 72 years old man is seen at the Emergency Department (ED) for a recent onset of dyspnea, cough and wheezing following what seems to be an upper airway viral infection. He had a past medical history of hypertension, benign prostatic hypertrophy and dyslipidemia. He took a diuretic and a statin. Two years ago, he was seen at the ED for what was considered a mild exacerbation of Chronic Obstructive Pulmonary Disease (COPD) and treated with inhaled bronchodilators and an antibiotic but had no measurement of expiratory flows on that occasion. He had stopped this medication due to fear of side effects. On questionnaire, he often had exercise intolerance and occasional symptoms of cough and wheezing at night or if exposed to respiratory irritants. He had a smoking history of about 8 pack/years. On arrival, his forced expiratory volume in 1 s (FEV_1_) was 45 % of predicted value (p.v.), with a FEV_1_/ forced vital capacity (FVC) ratio of 55. A few hours after receiving bronchodilators and oral corticosteroids, his FEV_1_ had improved to 70 % p.v. On discharge, he was offered a short-course of prednisone and started on a short-acting inhaled bronchodilator on demand plus a medium dose of inhaled corticosteroid. One month later, his FEV_1_ was 96 % p.v.

This case of asthma in an elderly patient, probably exacerbated by a viral respiratory infection illustrates common management problems in these patients such as confusion with chronic obstructive pulmonary disease (COPD) and poor patient report of chronic troublesome symptoms, often attributed to aging. It also shows the need to confirm the degree and reversibility of airway obstruction by objective means to confirm the diagnosis and offer an appropriate treatment. Careful record of symptoms patterns, smoking history, lung function tests and assessment of treatment response helps to assess the patient condition and treatment needs.

### Asthma is common and troublesome in the elderly

Asthma is a respiratory disease characterized by variable respiratory symptoms and airway obstruction associated with airway hyperresponsiveness (AHR) [1, 2]. These features are generally attributed to an airway inflammatory process with progressive bronchial remodelling [3, 4].

The prevalence of asthma in the elderly has been reported to be equal or even higher than in the general asthmatic population [5, 6]. As an example, the lifetime and current prevalence of asthma in patients over 65 years old was 10.6 and 7.0 % according to a study performed in the United States [7]. However, elderly patients have the highest asthma-related mortality, estimated at 51.3 million persons in USA in 2001 [8] (Table 1). Furthermore, they have a higher morbidity and rate of hospital admission for asthma than younger patients [6, 8–12]. This has been attributed to a frequent under- or mis-diagnosis, poor assessment and under-treatment of asthma in this group, but also related to many patients-related care gaps in the management of asthma in this population [6, 12–16]. Mortality rate from asthma is higher in the elderly than in younger patients (51.3 per million persons) of any other age group. Furthermore, many elderly asthmatic patients consider that their respiratory symptoms are normal, associated with aging and they often delay their consultation to a physician or to the ED, in addition to have many fears and misconceptions about their treatment [6, 17].

**Table 1:** Some characteristics of asthma in the elderly

Epidemiological	Increased morbidity and mortality Less frequent atopy
Clinical	Underdiagnosed and undertreated Asthma control more difficult to achieve Reduced perception of symptoms Difficulties to perform pulmonary function tests Numerous co-morbidities
Pathophysiology	Mixed (more neutrophilic airway inflammation) More severe airway obstruction Loss of lung elastic recoil – reduced respiratory muscles strength- small airways involvement Systemic inflammation and immunosenescence
Management problems	Insufficient understanding of the disease Memory impairment, hearing loss, poor sight Psycho-socio-economic problems
Therapy	Poor adhesion to therapy and follow-up Poor inhaler technique (arthritis, understanding…) Polypharmacy and increased risk of interactions
General	Poor nutrition, sedentarity, weight gain Reduced access to care

### How aging affects asthma pathophysiology

The elderly seems to suffer from a more severe phenotype of asthma, more resistant to the treatment, and with poorer outcomes. This remains however to be more adequately documented, as it can possibly change our approach to asthma management in this population. In this regard, aging is associated with a chronic low grade inflammation called inflamm-ageing [18]. Senescence can promote inflammatory (or down-regulate anti-inflammatory) cell genes, leading to a pro-inflammatory status. Since senescent cells have impaired replicative capacity, with time, this induces premature apoptosis or alters cell function.

Other factors may play a role in making asthma worse in the elderly. However little is known for example on the role of hormonal changes, obesity and metabolic changes. In this regard, although incidence of obesity and metabolic syndrome was not higher in postmenopausal asthma patients than controls, Aydin et al. found an impairment of glucose metabolism and altered adipokine levels in asthma patients. How this may affect asthma remains to be studied [19].

### Physiological changes in the aging lung: the “asthma in the elderly phenotype”

Age-related physiological changes may influence the asthma phenotype. We previously showed that airway obstruction was more marked in elderly asthmatics compared with healthy elderly or younger asthmatic subjects [20]. This may be due to the addition of a progressive “physiological” airway obstruction to the changes associated to asthma. The decline in lung function associated with aging is considered to result from a reduction in compliance and elastic recoil of the lung, in addition to a reduction in respiratory muscle strength while closing and residual volumes (RV) increase [20, 21]. Furthermore, the possibility of an increased prevalence of airway hyperresponsiveness (AHR) in the elderly asthmatic compared to younger subjects has been suggested although determinants of such increase in airway response may differ from younger patients [21, 22].

In the elderly, asthma is more neutrophilic than in younger asthmatic patients, whatever the severity of asthma [20, 23]. This may be related to aging itself, to respiratory infections, corticosteroid intake or other factors. A change in microbiome can play a role in this change in airway inflammatory pattern and even contribute to systemic inflammation [24]. Eosinophils are also found but their function seems reduced [25]. Total and specific IgE are reduced and atopy is considered less frequent but it still affects a large proportion of patients over 65 years old [6, 26, 27].

Furthermore, two main sub-phenotypes have been described in regard to asthma in the elderly according to the onset of symptoms [9]. ‘*Early-onset*’, starting in childhood or early adulthood, and ‘*Late*-*onset*’, starting after 40 years old, this last being associated with a faster lung function decline, less reversible airway obstruction and more severe asthma. We however need to know more about these two types of patients in regard to prognosis and to better guide the treatment [28].

### Assessment of asthma

Asthma is under-diagnosed in the aged patient as symptoms, are often underreported or misinterpreted, and asthma is often confounded with other diseases such as COPD and heart failure [5, 6]. Furthermore, a poor perception of asthma has also been reported in this population [29]. Although very old or physically impaired patients may have difficulties to perform lung function tests, those are much useful to assess properly their asthmatic condition. Some elderly patients may have difficulties to perform spirometry due to physical or cognitive limitations. Furthermore, they often have a certain degree of “physiological” irreversible airway obstruction and the results should be interpreted with caution. Indeed, a fixed FEV_1_/FVC ratio of less than 0.70 can lead to overdiagnosis of airway obstruction due to airway disease. The lower limit of normal values in uncertain in this age group. So, additional findings such as a significant improvement in FEV_1_ after bronchodilator (12 % and at least 200 ml) may support a diagnosis of asthma. In the presence of normal expiratory flows, a bronchoprovocation test (e.g. methacholine) may be ne necessary. Furthermore, even this is still debated, it has been suggested that airway hyperresponsiveness (AHR) is more common in the elderly but its determinants may be different [30, 31]. In a review of studies looking at AHR according to age, Scichilone et al. suggested that the data supported an increased prevalence of AHR in the elderly [32]. Furthermore, although it would be useful to know more about the determinants of AHR in this population. The most important seems to be a reduced lung function and a history of smoking, while atopy is an independent determinant.

### Comorbidities

The number of co-morbidities associated with asthma increases with age and some of those conditions, or the medications required to treat these last, can sometimes affect asthma control [33]. Gastro-oesophageal reflux, chronic rhino-sinusitis, obstructive sleep apnoea and heart conditions are common in the elderly [33, 34]. Obesity is common as we gain weight with age, making asthma control more difficult [35]. Asthma drugs, particularly oral corticosteroids, may also affect concomitant conditions such as osteoporosis, cataracts and glaucoma.

Not only could elderly asthmatics who smoke or have a past history of smoking have a change in their asthma phenotype, being more severe and more neutrophilic, but they may have developed the asthma-COPD overlap syndrome (ACOS) [1, 36]. In a study by Milanese et al., more than one-third of elderly asthmatic patients, despite receiving GINA step 3–4 antiasthmatic therapy, had an Asthma Control Test Score ≤19, a quarter experiencing at least one severe asthma exacerbation in the previous year [37]. One third of patients were classified as having ACOS due to the presence of chronic bronchitis and/or CO lung diffusion impairment and this group had poor asthma outcomes. A more recent study by de Marco et al. also showed that subjects with ACOS had an earlier age of asthma onset and the highest hospitalisation rate [38]. These observations may explain the global poorer outcome in elderly but other factors could also play a role.

### Treatment of asthma in the elderly

Asthma in the elderly is commonly under-assessed and treatment is often not in keeping with current guidelines [12, 39–43]. The elderly patient may have concerns about the side-effects of the medication, poor inhaler technique or insufficient inspiratory flows, and asthma management can be affected by memory impairment or economic difficulties [6, 12]. Insufficient adherence to therapy is also frequently reported and the above-mentioned problems, in addition to poor access to care, cultural specificities, or other personal characteristics may explain such reduced adherence [40, 41]. It is also possible that the elderly asthmatic is more resistant to therapy either due to a change in asthma inflammatory phenotype or airway functional changes. This remains to be further studied.

As elderly patients were usually excluded from randomized clinical trials on new treatments, there are few data on the optimal management of asthma in this population. It is therefore recommended to use the same guidelines as for the general population, hoping that further research will guide future recommendations [1, 6]. Control of symptoms, optimisation of pulmonary function, and prevention of future risks including exacerbations, while avoiding side-effects of the medication will guide our approach, which should be carefully individualized.

### Nonpharmacological treatment

This type of interventions include smoking cessation, influenza and pneumococcal vaccines. Furthermore, environmental control, promotion of active healthy lifestyle, with good dietary habits, and regular exercise should be promoted. Weight loss, in the presence of obesity, may markedly improve asthma but is often difficult to achieve [39].

Education of the patient and detection of management difficulties are mandatory [44, 45]. It has been demonstrated that with a more aggressive treatment and regular medical review, acute health care use could be reduced and quality of life improved in the elderly asthmatic patient [46]. A multidisciplinary approach has been proposed for this population, taking into account the various aspects of pharmacotherapy, education, psycho-socio-economical problems co-morbid conditions and acute care needs.

### Pharmacotherapy

As mentioned previously, the approach will be similar to the general population. Beta-blockers, mainly nonselective forms, are commonly used in elderly patients and may result in worsening asthma symptoms. Even topical forms for treatment of glaucoma have been implicated in severe bronchoconstriction [47].

Rapid-acting β2-agonists should be provided as rescue therapy, on demand, at the minimal dose and frequency. They are usually well tolerated but if they cause tremor or tachycardia, the dose should be reduced while maintenance therapy revised to minimize their need.

As in younger patients, inhaled corticosteroids (ICS) are the mainstay of asthma treatment in the elderly, although they are insufficiently used in this population [1, 40]. If insufficient to control asthma, we should first check inhaler technique, adherence, environmental factors, smoking status and co-morbid conditions. High-dose ICS and oral corticosteroids should be avoided whenever possible but if this is necessary over prolonged time-periods, prevention of bone loss by appropriate therapy is mandatory.

Associations of ICS and long-acting β2-agonists (LABA) such as formoterol or salmeterol are the first choice of treatment if a low-dose ICS is insufficient [1]. These treatments are usually well tolerated but side-effects, particularly if the patient is suffering from heart conditions should be documented. LABA should never be prescribed as monotherapy in asthma while they often are in COPD [1, 48]. Furthermore, budesonide/formoterol maintenance and reliever therapy has been shown to be an effective and well-tolerated treatment in elderly as in younger asthmatic patients [49].

Leukotriene receptor antagonists such as montelukast are safe in the elderly but we have few data on their usefulness in this group. Anti-cholinergic agents such as tiotropium have interesting properties for the elderly patients as response to this type of agents is well preserved with age particularly when asthma is severe or in the presence of side-effects of LABA [50].

Monoclonal antibodies such as omalizumab may be useful in severely asthmatic atopic patients whatever the age [51]. Theophylline is now rarely used, but if so, due to its potential for side-effects and risk of drug interactions, particularly in the aged patient, with frequent heart conditions and poly-pharmacy, its effects should be carefully monitored.

As asthma tends to be more severe and is associated with a high morbidity and increased mortality in the elderly patient, its treatment should be carefully selected and its effects checked regularly. As mentioned previously, as few studies have been performed specifically in this population, there is an urgent need to evaluate current and future therapies in this group. Reduced response to therapy and resistance to corticosteroids in the elderly may be another barrier to good control and adherence to therapy; this may result from a change in asthma phenotype but it remains to be better documented (Table 2).

**Table 2:** Research needed

• What are the effects of aging on lung and immune system • Better characterize the phenotype o Role of microbiome and infections o Role of environment o Influence of atopy, smoking, co-morbidities, gender • What are the consequences of recent vs. long-standing asthma • Determine what is the optimal management of asthma • How educate the elderly and develop an effective multidisciplinary approach • Should drug therapy be better targeted to this population o How is it different from younger patients? o What is the response to therapy (short and long-term outcomes)?	

## Conclusion

Asthma is common in the elderly and is often confounded with other conditions such as COPD or cardiac diseases. A change in asthma phenotype in the elderly and the many deficiencies observed in the management of this population may explain the poor outcomes observed. These care-gaps should be considered and more research performed on the optimal management of asthma in the aging patient.

**Table:** 

**Key**-**messages for the clinician** • Asthma in the elderly should be distinguished from other conditions such as COPD and heart conditions. • Careful assessment of asthma control and barriers to achievement of such control - particularly adherence to therapy, poor inhaler techniques, influence of co-morbidities and other management deficiencies - should be addressed. • As we have few data on the optimal management of asthma in this population, current general guidelines should be used. • A multi-disciplinary approach should be ensured to look at the various global aspects of elderly asthmatic health.	
